# Consistent individual differences in the social phenotypes of wild great tits, *Parus major*

**DOI:** 10.1016/j.anbehav.2015.07.016

**Published:** 2015-10

**Authors:** L.M. Aplin, J.A. Firth, D.R. Farine, B. Voelkl, R.A. Crates, A. Culina, C.J. Garroway, C.A. Hinde, L.R. Kidd, I. Psorakis, N.D. Milligan, R. Radersma, B.L. Verhelst, B.C. Sheldon

**Affiliations:** a Edward Grey Institute of Field Ornithology, Department of Zoology, University of Oxford, Oxford, U.K.; b Department of Anthropology, University of California, Davis, CA, U.S.A.; c Smithsonian Tropical Research Institute, Ancon, Panama; d Behavioural Ecology Group, Department of Animal Sciences, Wageningen University, Wageningen, The Netherlands; e Mathematical Institute, University of Oxford, Oxford, U.K.; f Evolutionary Ecology Unit, Department of Biology, Lund University, Lund, Sweden; g Department of Ecology and Genetics, Uppsala University, Uppsala, Sweden

**Keywords:** animal personality, *Parus major*, repeatability, social behaviour, socixal network analysis

## Abstract

Despite growing interest in animal social networks, surprisingly little is known about whether individuals are consistent in their social network characteristics. Networks are rarely repeatedly sampled; yet an assumption of individual consistency in social behaviour is often made when drawing conclusions about the consequences of social processes and structure. A characterization of such social phenotypes is therefore vital to understanding the significance of social network structure for individual fitness outcomes, and for understanding the evolution and ecology of individual variation in social behaviour more broadly. Here, we measured foraging associations over three winters in a large PIT-tagged population of great tits, and used a range of social network metrics to quantify individual variation in social behaviour. We then examined repeatability in social behaviour over both short (week to week) and long (year to year) timescales, and investigated variation in repeatability across age and sex classes. Social behaviours were significantly repeatable across all timescales, with the highest repeatability observed in group size choice and unweighted degree, a measure of gregariousness. By conducting randomizations to control for the spatial and temporal distribution of individuals, we further show that differences in social phenotypes were not solely explained by within-population variation in local densities, but also reflected fine-scale variation in social decision making. Our results provide rare evidence of stable social phenotypes in a wild population of animals. Such stable social phenotypes can be targets of selection and may have important fitness consequences, both for individuals and for their social-foraging associates.

## Highlights

• Social networks may be useful to quantify individual differences in sociality.
• We test this by measuring individual consistency in replicated social networks.
• A population of 729–1053 great tits was sampled a total of 41 times over 3 years.
• Individuals showed relatively high consistency in social network position.
• Controlling for space use found individual variation in fine-scale social behaviour.

The application of social network analysis to animal populations has become an important component of the behavioural ecologist's toolbox, leading to novel insights into the potential costs and benefits of sociality. In particular, different social positions within animal groups can have associated advantages and disadvantages, including for social information use (Aplin, Farine, Morand-Ferron, & Sheldon, 2012), disease transmission (Godfrey, Bull, James, & Murray, 2009; Hamede, Bashford, McCallum, & Jones, 2009), mate choice (Oh & Badyaev, 2010), competition (D. Farine & Sheldon, 2015) and long-term reproductive success (Mcdonald, 2007). It therefore seems likely that social network position may be under selection; further, that there may be trade-offs associated with differing social behaviours (e.g. central individuals receive better social information, but are also more susceptible to diseases). However, while some studies have found some aspects of network position to be heritable (Fowler, Dawes, & Christakis, 2009; Lea, Blumstein, Wey, & Martin, 2010), surprisingly little is known about whether individuals are repeatable and consistent in their social network characteristics. Establishing whether individuals have consistent social phenotypes is important for understanding the degree of plasticity in social behaviour, can set an upper limit to heritability, and is an important first step in understanding the significance of social network structure for individual fitness outcomes.

Individual level variation in behaviour can be understood within the context of the animal personality literature, which focuses on broad trait categories including boldness, exploration behaviour, risk taking, aggression and sociability (Bell, Hankison, & Laskowski, 2009; Reale, Reader, Sol, McDougall, & Dingemanse, 2007). Consistent within- and between-individual differences have been extensively documented for exploration behaviour and boldness by assaying individuals in isolation (Bell et al., 2009; Dingemanse et al., 2012) and by using dyadic interactions for traits such as aggression (Blumstein, Petelle, & Wey, 2013). In contrast, individual variation in sociality has been quantified in a more limited range of studies and usually investigated with assays of shoaling tendency, separation tests or group size choice (Cote & Clobert, 2007; Cote, Fogarty, & Sih, 2012; Harcourt, Sweetman, Johnstone, & Manica, 2009; Mills & Faure, 2000; Reale et al., 2007). Social networks provide a new opportunity by which to study sociability in a complex social context, with a set of standardized, well-understood metrics (Sih, Bell, & Johnson, 2004; Sih, Cote, Evans, Fogarty, & Pruitt, 2012).

Individual variability in social behaviour may also have implications for the evolution and maintenance of other personality traits, for example if individuals adopt stable social roles that are fine-tuned through positive feedback loops or frequency dependence (Bergmuller & Taborsky, 2010; Wolf, van Doorn, Leimar, & Weissing, 2007). A relationship between social behaviour, social structure and other individual level behavioural traits has been found in fish (Croft et al., 2009; Pike, Samanta, Lindstrom, & Royle, 2008; Schurch, Rothenberger, & Heg, 2010), sleepy lizards, *Tiliqua rugosa* (Godfrey, Bradley, Sih, & Bull, 2012) and great tits (Aplin et al., 2013; Snijders et al., 2014). In great tits, birds with higher scores for the personality trait ‘exploration behaviour’ are more central in winter and spring social networks (Aplin et al., 2013; Snijders et al., 2014). Aplin, Farine, Mann, and Sheldon (2014) tested how these individuals made social decisions when flocking, and found that fast explorers used less social information and showed less social cohesion, suggesting a mechanism by which broad-scale differences in social structure may emerge (also see Boogert, Farine, and Spencer (2014)).

An assumption of individual consistency in social behaviour is therefore important for a range of questions in studies of social networks and animal personality. Yet networks are rarely repeatedly sampled, with studies often drawing inferences about social structure and dynamics despite little or no replication. Two exceptions include recent studies in wild yellow-bellied marmots, *Marmota flaviventris* (Blumstein et al., 2013) and captive spotted catsharks, *Scyliorhinus canicula* (Jacoby, Fear, Sims, & Croft, 2014). In Blumstein et al. (2013) a moderate repeatability was found for a measure of aggressive interactions, with social networks measured over several years (*R* = 0.22), but there was no relationship between social aggression and the individual level personality trait of defensive aggression. Jacoby et al. (2014) measured social associations in replicated groups of captive juvenile sharks, quantifying repeatability across different habitat treatments. Network strength was repeatable (*R* = 0.46), with little plasticity across treatments, and largely driven by stable individual level preferences for aggregating in specific group sizes. The authors suggested that this consistent behaviour provided evidence for social personality types in sharks; however, the relatively short-term nature of the measurements (14 days) limited broader conclusions.

Here we investigated social behaviour in a PIT-tagged population of great tits over three winters. This large-scale study provided a unique opportunity to assess individual consistency in social network position over both short-term (week to week) and longer-term (between years) timescales, by using a grid of feeding stations fitted with RFID antennae to capture ‘snapshots’ of the spatiotemporal flocking patterns of a population of 729–1053 individuals. We first collected 13–14 replicated foraging social networks for each year, and measured individual repeatability in network attributes within each winter season. Second, we combined within-year sampling periods to construct a foraging social network for each winter, and measured between-year individual repeatability in the same network attributes. Repeatabilities were compared across age and sex classes. Finally, we compared our repeatability estimates to those calculated from permutations that controlled for the spatial location of individuals in each sampling period. This approach corrected for any potentially confounding interaction between social networks and spatiotemporal differences in local population density (Farine et al., 2015). This allowed us to identify the relative contributions to social network position of spatial influences (including dispersal, settlement and movement decisions (Cote & Clobert, 2007; Cote, Fogarty, Weinersmith, Brodin, & Sih, 2010), and more fine-scale variation in social decision making.

## Methods

### Study Site and Species

The study was conducted over 3 years (December 2011–March 2014) in a population of great tits in Wytham Woods, Oxfordshire, U.K. (51°46′N, 01°20′W). This population has been the subject of a long-term breeding survey since its establishment in 1947, with 1018 great tit nestboxes installed throughout the core area. The provision of artificial nestboxes allows birds to be trapped as nestlings and breeding adults, with trapped birds fitted with both a metal leg ring from the British Trust for Ornithology and a plastic leg ring containing a uniquely identifiable passive integrated (PIT) tag from IB Technology, Aylesbury, U.K. Birds were aged and sexed upon capture using either previous breeding records or plumage coloration. Over winter, great tits form loose fission–fusion flocks of unrelated individuals, with groups moving between patchy and ephemeral food sources, including bird feeders. In this period, mist netting at regular intervals targeted birds immigrating into the population, such that the large majority of wintering individuals were ringed and PIT-tagged. See Aplin et al. (2013) and Matechou, Cheng, Kidd, and Garroway (2015) for a formal analysis of what percentage of the population was tagged in winter, estimated at over 90% in 2011–2012.

### Field Observations

**Figure 1:**
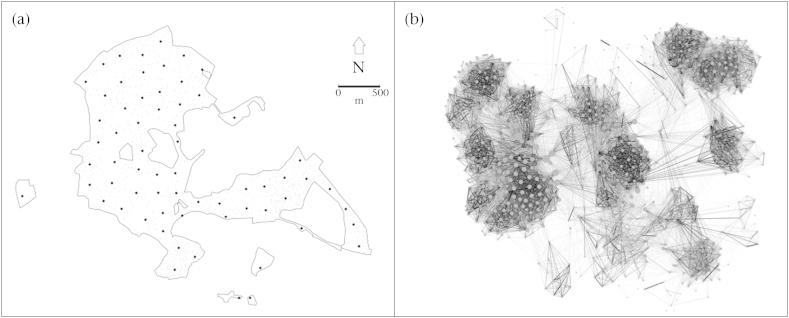
(a) Map of the study site showing the location of 65 feeding stations, each approximately 250 m apart and opening to scan for PIT-tagged great tits for 26–28 days of data collection over each of three winters. Smaller points on the map show the 1018 artificial nestboxes installed in the woodland. (b) An example of a social network constructed using this information on spatiotemporal foraging behaviour; the network is shown for the entire 2013–2014 winter period. Each node is one of 816 individuals and links between nodes are scaled between 0 (never observed in the same foraging flock) to 1 (always observed in the same foraging flock). Node size is scaled by unweighted degree (1–226).

Data were collected in three winter seasons: from 3 December 2011 to 27 February 2012 (Year 1), 1 December 2012 to 3 March 2013 (Year 2) and 30 November 2013 to 2 March 2014 (Year 3). Bird feeders were filled with unhusked sunflower seed and deployed at 65 locations, each approximately 250 m apart throughout the study site (Fig. 1a). These feeding stations opened from before dawn to after dusk for 2 consecutive days in every 7, resulting in 26 days of data collection over 13 sampling periods in the first winter (2011–2012) and 28 days of data collection over 14 sampling periods for the second and third winters (2012–2013, 2013–2014). Each feeding station had two access points fitted with a radio frequency identification (RFID) antenna and a data-logging device that scanned for PIT tags every 16th of a second. When great tits landed on the feeding station, their unique 10-digit hexadecimal PIT-tag code was registered and recorded on the data-logging hardware with an associated time and location, providing detailed spatiotemporal snapshots of individual foraging behaviour.

### Social Networks

In each season, simultaneous sampling thus captured the weekly flocking choices of foraging individuals. We used a Gaussian mixture model to detect clusters of visits in these spatiotemporal data streams (Psorakis, Roberts, Rezek, & Sheldon, 2012). This method detects high-density periods of feeding activity (gathering events) without imposing subjective and artificial assumptions about the temporal boundaries of groups. This provided data on the identity of birds in each group, and was used to calculate an average group size for each individual in each sampling period and over the winter season. Social associations were assigned based on their presence in the same gathering event, similar to a gambit of the group approach (Franks, Ruxton, & James, 2010; Whitehead & Dufault, 1999). Association strengths for each dyad were calculated using the simple ratio index, where associations are scaled between 0 (never observed in the same foraging group) to 1 (always occurred in the same foraging group) (Cairns & Schwager, 1987). This approach is consistent with previous work on this population (see Aplin et al., 2015; Aplin et al., 2013; Farine, Garroway, & Sheldon, 2012). Social networks were then created for each sampling period, resulting in a total of 41 networks, containing an average of 561 (409–851) individuals. Social networks were also created for each winter season, summing data from all within-season sampling periods to create three networks containing 1053 (Year 1), 729 (Year 2) and 816 (Year 3) individuals (e.g. see Fig. 1b). Network construction and analysis were performed in R v.3.1.1 (R Core Team, 2012) using the asnipe package (Farine, 2013).

In addition to average group size, three social network metrics were calculated for each individual in each sampling period and season: unweighted degree, overall association strength (weighted degree) and betweenness. These metrics respectively measure: (1) the number of conspecifics with which the focal individual was observed foraging, giving an idea of overall gregariousness (see Fig. 1b, in which nodes are scaled by unweighted degree); (2) the total interaction rate for the focal individual with all other individuals, representing a focal measure of individual sociability; and (3) the number of shortest paths from all individuals to all other individuals that pass through the focal individual, important for the spread of information and disease (Croft, James, & Krause, 2008). Clustering coefficient was also calculated for individuals for each winter; this metric requires extensive observations of group membership to show interindividual variation and could not be reliably calculated for within-season sampling periods. It is representative of the extent to which nodes tend to cluster together to form distinct cliques, indicating how ‘tight-knit’ individuals are in their groups. All social network analyses were done in R packages sna (Butts, 2008).

### Statistical Analysis

We assessed both within-year and between-year variation in social phenotypes by calculating individual level repeatability in social network metrics and group size. Repeatability is a measure of the total variation that is reproducible among repeated measures of the same individual (Nakagawa & Schielzeth, 2010), giving an indication of the consistency of individual phenotypes. Yet network metrics are not independent of other individuals in the social network, and will vary with sampling effort, population size and density. We first dealt with these possible sources of error by collecting the data for each repeated network in the exact same way, with the same sampling intensity and effort. Second, we calculated repeatability in two ways. A linear mixed-effects model (LMM) was used to calculate the intraclass correlation coefficient (ICC), with repeatability adjusted to account for population size, network density and date of each repeated measure (Nakagawa & Schielzeth, 2010). The square root of each measure was taken, except for betweenness, which was modelled as an exponential function. This normalized the distributions, and repeatability was estimated from the variance of the individual random effect divided by the sum of the individual-level variance and the variance of the random error. Repeatability significance was estimated with Markov chain Monte Carlo sampling using restricted maximum likelihoods and default priors in the R package MCMCglmm (Hadfield, 2010). This method has the advantage of being easily comparable with most previous studies (e.g. see Bell et al., 2009), but should be interpreted with caution for network measures with a more global scope, such as betweenness. Using this method, repeatability estimates were further compared across age and sex classes. Individuals were aged (adult/first year) and sexed (male/female) using either previous breeding records or plumage coloration. Differences between classes were determined by calculating pairwise differences in *Z*-transformed repeatability estimates and assessing whether confidence intervals, CI, overlapped with zero (Nakagawa & Schielzeth, 2010).

We also calculated repeatability using methods described in Wilson, Krause, Dingemanse, and Krause (2013), with a null model based on repeated node-based randomizations of the networks. This test explicitly controls for the nonindependence of data within networks, and compares the sum of the variances for individuals' network positions across observed networks (SV_O_) to the sum of individual level variances in position from randomized networks SV_R_ (Wilson et al., 2013). Individuals were ranked within each network and scaled between 0 and 1. Individual network positions were thus relative to all others in that network, with small values of SV_O_ indicating a similar relative ranking across all repeated samples. Significance was determined by comparing the SV_O_ for each network measure to a frequency distribution of SV_R_ values generated from 10 000 node randomizations of observed data.

A component of an individual's social phenotype might simply result from choices about where and when to forage. For example, if individuals differ in their propensity to settle in areas of different density, then, given that population density is expected to influence many aspects of social networks, a component of the interindividual variation will result from such individual settling decisions. To identify the influence of such spatial effects, we designed a spatiotemporal null model that aimed to estimate the expected individual repeatability if individuals simply differed consistently in their occurrence at different feeding locations, but were not repeatable in their behaviour within these locations. To create this null model we carried out 1000 permutations of each network that maintained which locations (feeders) and time periods (weekends) each individual was recorded, but randomized their social phenotype within these spatiotemporal choices. In each permutation, each individual was assigned to one of the locations where it was observed, where the probability of being assigned to each location was generated by their activity at each one (number of flocks they were observed in). On the majority of occasions (65%), individuals were only present at one location on a given weekend.

Following this assignment, the identity of individuals within the same locations during the sampling period was randomly swapped, so that each individual adopted the phenotype of another individual occurring at that feeder that weekend, Thus, in each permutation, individuals were assigned a new social phenotype (network metric). This permutation procedure maintained the structure of the data and the same variation in network metrics, but removed the link between observations of the same individual across multiple replicates. This represents a null model in which the effects of individual locations in space and time (at the scale of weekend samples) are retained, but no other individual differences. Estimation of repeatability (using the LMM approach) was then drawn from each permuted data set, and the 95% range of these estimations illustrated the expected range of repeatability if individuals just differed in their spatiotemporal occurrence, but not in their social choices.

### Ethical Note

All work was subject to review by the local ethical review committee at the Department of Zoology (University of Oxford) and also adhered to U.K. standard requirements. The work was conducted as part of a larger ongoing research project at Wytham woods, and all birds were caught, tagged and ringed by appropriate BTO licence holders.

## Results

**Figure A1:**
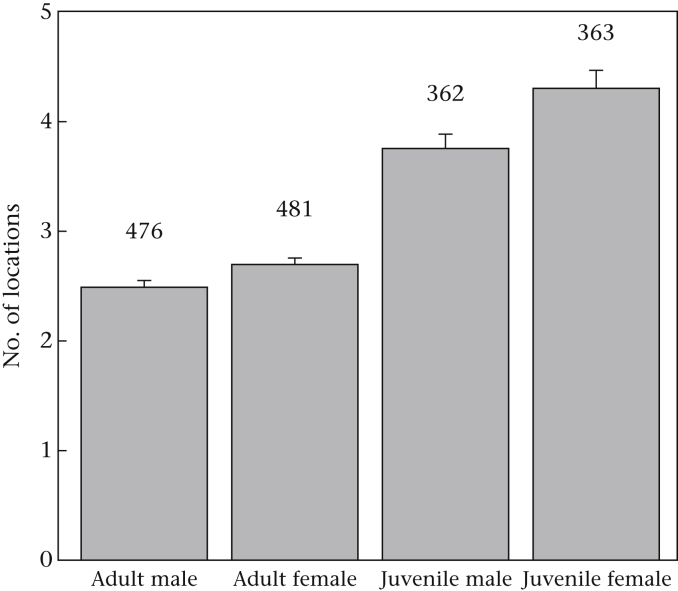
The average number of locations per year where individuals of the different age and sex classes were observed foraging. Lines show SEs and data are used from three winter seasons. Numbers show the number of unique individuals recorded in each class.

Within each winter season, individuals were observed in a median of 11 (Year 1), 10 (Year 2) and 12 (Year 3) sampling periods, with 9835 (Year 1), 6853 (Year 2) and 7940 (Year 3) measures derived from 1053 (Year 1), 729 (Year 2) and 816 (Year 3) individuals. Over each full winter, the median range encompassed three feeding stations with eight moves between feeders (also see Aplin et al., 2013; Fig. 1a); there was no difference in the distance or type of spatial movements undertaken by males and females, but first-years moved more than adults (see Appendix Fig. A1).

**Figure A2:**
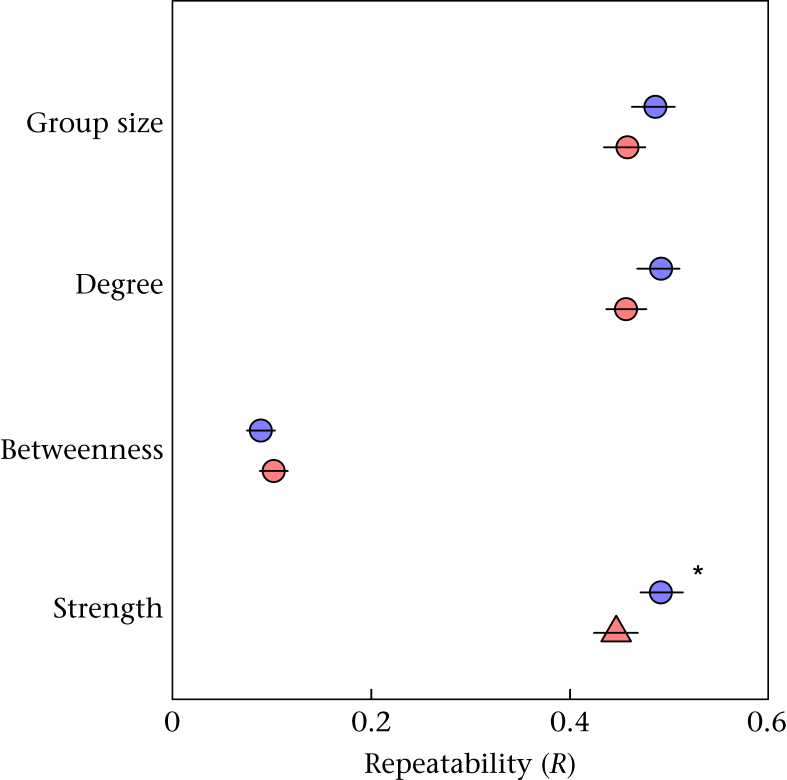
The repeatability measures for the different sex classes, showing the 95% confidence interval for each measure. Nonoverlapping confidence intervals at the 95% level are marked with an asterisk. Group size and degree measures do not overlap at the 84% confidence interval (nonoverlapping 84% confidence intervals are equivalent to a *z*-test at the 0.05 level). All 41 repeated social networks across 3 years were used to calculate repeatability estimates, additionally controlling for year (1–3). Blue nodes are males; red nodes are females.

**Figure 2:**
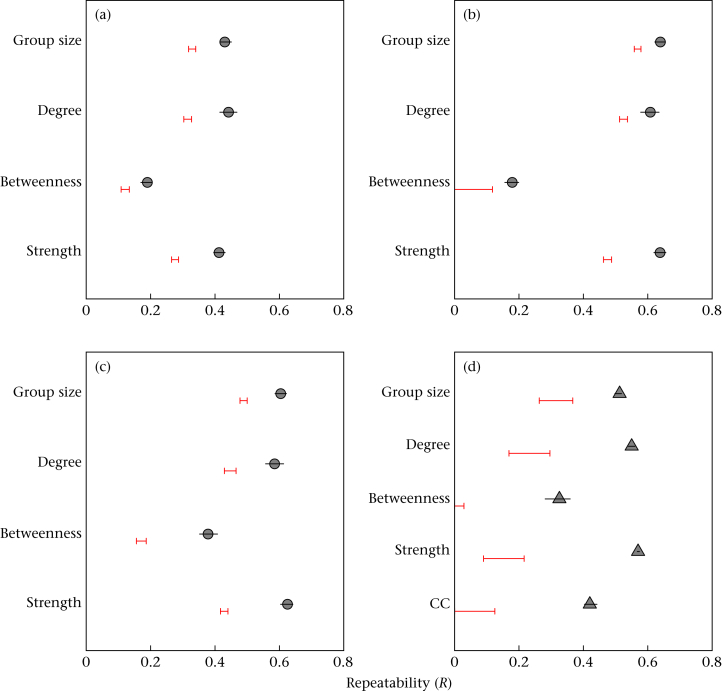
Repeatabilities and 95% confidence intervals for average group size and four social network metrics: degree, betweenness, association strength and clustering coefficient (CC; between years only for the latter). Results are shown for three winter data collection periods (within-season repeatability): (a) 2011–2012 winter; (b) 2012–2013 winter; (c) 2013–2014 winter. (d) Results compared across 3 years (between-year repeatability). Estimates whose confidence intervals do not cross 0 (*y*-axis) are significantly repeatable at the α = 0.05 level. Horizontal red lines show 95% range of the repeatability estimates calculated from 1000 data randomizations controlling for spatial location.

Birds were significantly consistent in all measured social behaviours. In all years, group size (Year 1: *R* = 0.43; Year 2: *R* = 0.64; Year 3: *R* = 0.60), degree (Year 1: *R* = 0.46; Year 2: *R* = 0.61; Year 3: *R* = 0.58) and association strength (Year 1: *R* = 0.41; Year 2: *R* = 0.64; Year 3: *R* = 0.63) were moderately to highly repeatable. Betweenness (Year 1: *R* = 0.19; Year 2: *R* = 0.18; Year 3: *R* = 0.38) was less consistent, with a higher variability (Fig. 2). Males and adults were significantly more repeatable than females and birds in their first year in all measures except betweenness, for which the results were less consistent (Tables 1 and 2). However, the differences in absolute repeatabilities were small, ranging between 0.01 and 0.14 for age differences and between 0.01 and 0.06 for sex. Furthermore, the 95% CIs overlapped for all but one metric (see Appendix Fig. A2), suggesting that the biological importance of these differences may be relatively minor compared to the overall effect of individual differences in social phenotype. Population size, network density and date were included as fixed effects in all models. Population size and network density both had a significant effect in all but two models, but date was less consistent (see Appendix Tables A1 and A2). Finally, when the repeated within-year networks were analysed using a network randomization method (Wilson et al., 2013), the results for network metrics were similar, with significant repeatability in all metrics, but with highest repeatability in degree centrality and association strength (see Table 3).

**Figure A3:**
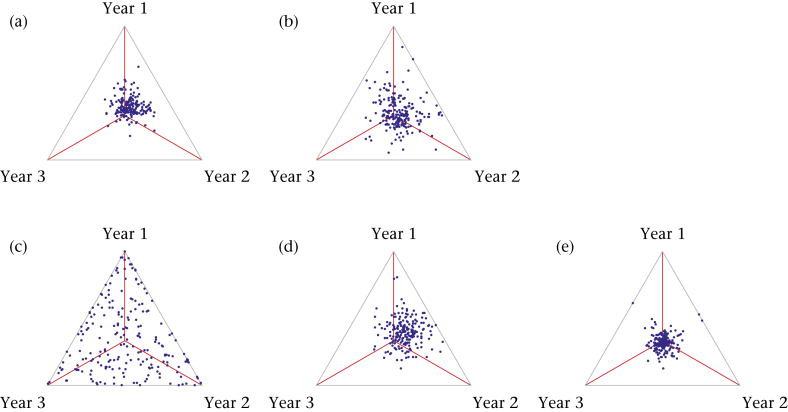
Simplex plots of repeatabilities for 210 individuals observed over three winter seasons of social network data collection. Each point represents one individual, and a perfect correlation between all three seasons is the central intercept of axes. Data are scaled between 0 and 1 within each season. Five winter-long measures of social behaviour are shown: (a) average group size, (b) unweighted degree, (c) betweenness centrality, (d) association strength and (e) clustering coefficient.

**Figure 3:**
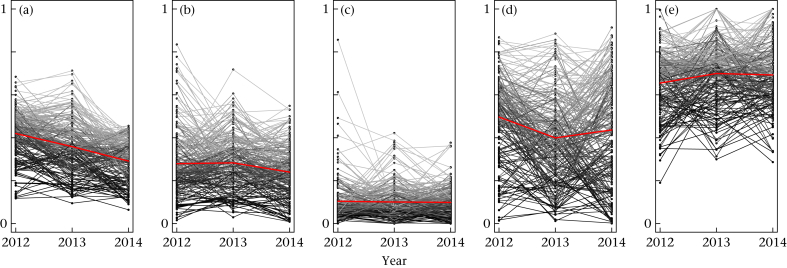
Repeatabilities for 210 individuals observed over three winter seasons of social network data collection. Scores are scaled between 0 and 1 for all individuals within each season. Points are individuals and grey lines connect their scores in each year. Red lines show the average score for all individuals in each year. Five winter-long measures of social behaviour are shown: (a) average group size (*R* = 0.51), (b) degree (*R* = 0.55), (c) betweenness centrality (*R* = 0.33), (d) strength (*R* = 0.57) and (e) clustering coefficient (*R* = 0.43).

It was also possible to calculate individual consistency in social behaviour between years, with 565 great tits observed in at least two winters, and 210 individuals observed in all 3 years. Similarly high repeatabilities were observed for degree (*R* = 0.55, 95% CI 0.54–0.56), group size (*R* = 0.51, 95% CI 0.50–0.52), association strength (*R* = 0.57, 95% CI 0.567–0.574) and clustering coefficient (*R* = 0.43, 95% CI 0.41–0.44; Fig. 3). Estimated repeatability for betweenness was slightly lower, with a higher variance (*R* = 0.33, 95% CI 0.28–0.36; Fig. 3, also see Appendix Fig. A3 for an alternative visualization). Again, results were similar for the network metrics when using the test statistic SV_O_, with significant repeatability for all measures, but with the highest repeatability in degree and strength (see Table 3).

For both within- and between-year estimates of repeatability, the four measures of social behaviour were compared to repeatability estimates calculated after the data were randomized within sampling period and within location. This spatially constrained null model explained variable amounts of the individual repeatability for different phenotypes. For example, within years, it accounted for only 1–19% of the within-individual consistency in betweenness centrality, but 35–55% of the consistency in an individual's average group size. This pattern was reflected in the between-year individual consistency, with very little of the repeatability in betweenness centrality due to spatial location, but with 32% of repeatability in average group size due to location. Yet in all cases, the repeatability estimate calculated from the observed data was higher than the 95% range of those generated from the spatially constrained permuted data sets, suggesting that local variation in social phenotypes contributed significantly to within-individual repeatability in social network position (Fig. 2).

## Discussion

When sampled throughout three winter seasons, individual great tits were repeatable in their social behaviour and social network phenotype. In two recent studies, the mean repeatability of the network metric ‘strength’ was 0.22 in yellow-bellied marmots (Blumstein et al., 2013) and 0.46 in captive spotted catsharks (Jacoby et al., 2014). This measure had a repeatability in our system of *R* = 0.41–0.62 depending on the year analysed, suggesting that great tits have a comparatively high consistency in social behaviour. A recent meta-analysis of behavioural consistency across various taxa also placed the average repeatability of all behaviour at 0.37 (Bell et al., 2009), indicating that individual great tits were relatively highly consistent in their group size choice (*R* = 0.45–0.65) and gregariousness (degree: *R* = 0.46–0.63). Repeatability was more moderate for the social network measures of betweenness (*R* = 0.18–0.38). Overall, when taken in combination with previous studies, these results suggest that network metrics tend to be consistent at the individual level. This is of fundamental importance for our understanding of the interaction between individual behaviour and social structure in animal social networks.

When within-individual consistency was measured across 3 years using winter-long social networks, similarly high repeatability measures were observed for group size (*R* = 0.51), degree (*R* = 0.55) and association strength (*R* = 0.57). Significant repeatability was further observed in clustering coefficient (*R* = 0.43) and betweenness (*R* = 0.33). That repeatability estimates tended to be higher when measured across years, as opposed to when measured within years, may indicate that winter-long social networks may provide the most accurate estimate of individual social phenotypes in this system, or alternatively that the most consistent individuals are more likely to survive from year to year; these hypotheses need further investigation to disentangle. Overall, however, our results demonstrate that social behaviour is stable over both short and long timescales.

Such within- and between-individual variation in social phenotypes may have important implications for the evolution and ecology of sociality. More central network positions have been shown to be associated with various benefits, including social information transfer (Aplin et al., 2012) and reproductive success (Mcdonald, 2007; Oh & Badyaev, 2010), but may also expose social individuals to higher rates of competition (Oh & Badyaev, 2010) and disease (Christley et al., 2005). It thus seems likely that there is functional significance to variation in social network position, with life history trade-offs leading to the evolution of consistent individual differences in social behaviour (Dall, Houston, & McNamara, 2004; Krause, James, & Croft, 2010). Given that repeatability can indicate the upper limits to heritability, our results further suggest that individual great tits could show heritable differences in sociality, an avenue that merits further study.

While territoriality is low over the winter period, our woodland study site varies in microhabitat quality and in local population density. It is thus likely that some of the consistency in social behaviour is related to individual differences in space use (Shizuka et al., 2014). Such differences may also explain the slightly higher repeatability of males and adults, which tend to make fewer spatial movements than females and first-years, although this is also consistent with across-species patterns of behavioural repeatability (Bell et al., 2009). We applied spatially constrained randomizations to disentangle these effects and showed that while some individual differences could be explained by spatial location, an additional component of social behaviour was driven by within-location flock membership choices. However, habitat choices might also be a reflection of social phenotypes. Tits use social information from conspecifics when making dispersal and settlement decisions (Nocera & Forbes, 2010; Nocera, Forbes, & Giraldeau, 2006; Pajero, White, & Danchin, 2007; Parejo et al., 2008). Individuals could potentially differ in this decision making based on individual differences in sociability (Blumstein, Wey, & Tang, 2009; Cote, Clobert, Brodin, Fogarty, & Sih, 2010), as observed in common lizards, *Lacerta vivipara* (Cote & Clobert, 2007) and mosquito-fish, *Gambusia affinis* (Cote, Fogarty, et al., 2010), in which more asocial individuals tend to stay in low-density patches and disperse when local population sizes become high. Future work could potentially explore these interactions with a combination of captive and wild translocation experiments, measuring individual variation in social behaviour across different contexts.

In summary, our study provides one of the few demonstrated examples of consistency in social network position, with birds maintaining relatively stable group sizes and network metrics over multiple timescales. This variation in social behaviour was partly attributable to individual differences in spatial location and space use, but a significant amount of between- and within-individual variation remained even when controlling for these factors. We can therefore identify individual social phenotypes, a vital first step in showing the adaptive significance of social network structure in wild animals. Future work should aim to quantify the potentially diverse fitness consequences associated with different social phenotypes, and further investigate how such selection can shape the evolution and ecology of animal social networks.

## Figures and Tables

**Table A1:** Outputs of linear-mixed models showing fixed effects for four network measures in each of three winter seasons

Year	Metric	Term	Estimate	Lower 95% CI	Upper 95% CI	*P*
2011–2012	Group size	PS	0.0005600	0.0003021	0.0008281	<0.001
ND	4.5827078	0.8800015	9.1275491	0.04
Date	−0.0179925	−0.0242837	−0.0106679	<0.001
Degree	PS	0.004594	0.003924	0.005316	<0.001
ND	58.828297	47.716612	68.944029	<0.001
Date	−0.013392	−0.031308	0.003472	0.150
Betweenness	PS	−0.4221	−0.5185	−0.3003	<0.001
ND	−1438.6215	−3161.8210	227.9183	0.098
Date	1.5072	−1.2775	4.3902	0.282
Strength	PS	0.0005202	0.0002851	0.0008156	<0.001
ND	0.6342989	−3.4182792	4.3784652	0.744
Date	−0.0141429	−0.0206190	−0.0076515	<0.001
2012–2013	Group size	PS	−0.0001.661	−0.0003.982	0.0007.208	0.166
ND	12.65	10.52	14.76	<0.001
Date	0.0000330	−0.003750	0.003693	0.984
Degree	PS	0.004958	0.004346	0.005538	<0.001
ND	38.830534	33.520697	43.934455	<0.001
Date	0.004751	−0.004275	0.014303	0.330
Betweenness	PS	−1.416	−1.600	−1.237	<0.001
ND	−6398	−8086	−4641	<0.001
Date	−0.0003055	−2.729	3.064	0.988
Strength	PS	0.0000085	−0.0002321	0.0002308	0.916
ND	10.33	8.006	12.29	<0.001
Date	0.002167	−0.001562	0.005576	0.238
2013–2014	Group size	PS	0.0005122	0.00000268	0.000933	0.026
ND	14.8	11.79	17.78	<0.001
Date	0.008140	0.005077	0.01103	<0.001
Degree	PS	0.004869	0.003660	0.006124	<0.001
ND	58.437816	50.106302	67.543610	<0.001
Date	0.000447	−0.007223	0.008739	0.892
Betweenness	PS	−0.2347	−0.4745	0.01216	0.060
ND	−2178	−3952	−425.4	0.012
Date	0.8131	−0.8573	2.431	0.338
Strength	PS	0.0007248	0.0003058	0.001189	<0.001
ND	12.12	9.476	15.29	<0.001
Date	0.00991	0.007116	0.0129	<0.001

Three fixed effects are included in each model: total population size (PS) for each sample, total network density (ND) for each sample and the date of each sample. Individual identity was included as a random effect in all models.

**Table A2:** A comparison of repeatability scores in three seasons of data collection

Model variation	Network metric	Year 1: 2011–2012	Year 2: 2012–2013	Year 3: 2013–2014
As reported in paper	Average group size	0.430	0.639	0.605
Degree	0.456	0.625	0.585
Betweenness	0.189	0.179	0.376
Strength	0.413	0.639	0.624
Including sample size for each individual	Average group size	0.428	0.631	0.601
Degree	0.440	0.592	0.579
Betweenness	0.481	0.138	0.327
Strength	0.409	0.606	0.590
Removing individual with <50 observations	Average group size	0.455	0.644	0.597
Degree	0.450	0.618	0.558
Betweenness	0.520	0.173	0.487
Strength	0.409	0.614	0.574

The results from three model variations are presented: (1) repeatability estimates as reported in the paper, with a linear mixed model including individual identity as a random effect and population size, network density and date as fixed effects; (2) the linear mixed model additionally including the number of observations for each individual on each weekend sampling period as a fixed effect; (3) the linear mixed model as above, but removing all individuals with fewer than 50 observations.

**Table 1:** Differences in the repeatability of social behaviour by sex

Metric	*N*(*N*_o_, *N*_i_) male	*N*(*N*_o_, *N*_i_) female	*R*_male_	*R*_female_	Effect size	Trend
Group size	10906 (706)	10186 (735)	0.48	0.45	0.83 (0.77,0.89)	M>F
Degree	10906 (706)	10186 (735)	0.49	0.45	0.95 (0.89,1.02)	M>F
Strength	10906 (706)	10186 (735)	0.49	0.44	1.58 (1.48,1.68)	M>F
Betweenness	10906 (706)	10186 (735)	0.08	0.09	0.60 (0.55,0.66)	F>M

M: male; F: female. All 41 repeated social networks across 3 years were used to calculate repeatability estimates for sex, additionally controlling for year (1–3). Differences are considered significant when 95% CIs for effect sizes do not overlap with zero. Sample size (*N)* shows both total number of observations (*N*_o_) and number of individuals (*N*_i_), and effect size shows 95% CIs.

**Table 2:** Differences in the repeatability of social behaviour by age class

Metric	*N*(*N*_o_, *N*_i_) Fy	*N*(*N*_o_, *N*_i_) Ad	*R*_Fy_	*R*_Ad_	Effect size	Trend
Group size						
Year 1	4441 (503)	5318 (514)	0.40	0.45	1.11 (1.02, 1.19)	Ad>Fy
Year 2	1237 (152)	5579 (573)	0.63	0.64	0.19 (0.13, 0.25)	Ad>Fy
Year 3	4016 (405)	3924 (411)	0.59	0.61	0.44 (0.37, 0.52)	Ad>Fy
Degree						
Year 1	4441 (503)	5318 (514)	0.42	0.47	1.11 (1.03, 1.19)	Ad>Fy
Year 2	1237 (152)	5579 (573)	0.60	0.63	0.51 (0.44, 0.57)	Ad>Fy
Year 3	4016 (405)	3924 (411)	0.54	0.64	1.60 (1.47 1 .73)	Ad>Fy
Strength						
Year 1	4441 (503)	5318 (514)	0.38	0.43	1.69 (1.56, 1.81)	Ad>Fy
Year 2	1237 (152)	5579 (573)	0.61	0.65	1.74 (1.61, 1.86)	Ad>Fy
Year 3	4016 (405)	3924 (411)	0.60	0.65	1.78 (1.62, 1.93)	Ad>Fy
Betweenness						
Year 1	4441 (503)	5318 (514)	0.54	0.46	−1.0 (−1.08, −0.92)	Fy>Ad
Year 2	1237 (152)	5579 (573)	0.17	0.18	0.14 (0.06, 0.21)	Ad>Fy
Year 3	4016 (405)	3924 (411)	0.37	0.45	1.99 (1.80, 2.18)	Ad>Fy

Age categories can change from first year (Fy) to adult (Ad) for individuals between years, so each year is presented separately (13–14 sampling periods). Differences are considered significant when 95% CIs for effect sizes do not overlap with zero. Sample size (*N)* shows both total number of observations (*N*_o_) and number of individuals (*N*_i_), and effect size shows 95% CIs.

**Table 3:** The observed (O) and randomized (R) sum of the variances (SV) of individual social position in the repeatedly sampled networks

Group	Metric	No. of samples	SV_O_	*S*_R_	*P*
2011–2012	Degree	13	50.24	83.86 (80.75, 87.77)	<0.001
Strength	13	51.43	83.94 (82.18, 85.73)	<0.001
Betweenness	13	69.02	83.85 (80.19, 87.31)	<0.001
2012–2013	Degree	14	22.29	55.65 (53.62, 57.96)	<0.001
Strength	14	21.61	55.73 (54.42, 57.13)	<0.001
Betweenness	14	46.54	55.09 (52.93, 57.85)	<0.001
2013–2014	Degree	14	28.80	61.81 (58.8, 65.13)	<0.001
As. Strength	14	25.84	61.83 (60.21, 63.52)	<0.001
Betweenness	14	48.82	61.74 (58.88, 65.34)	<0.001
Between-year	Degree	3	22.93	46.98 (40.57, 54.2)	<0.001
Strength	3	21.06	47.05 (43.30, 50.94)	<0.001
Betweenness	3	30.30	46.96 (40.61, 46.97)	<0.001
Clustering C.	3	28.19	49.52 (41.99, 56.33)	<0.001

Smaller values of SV indicate a higher individual repeatability in network metrics, and mean and 95% range are shown from 10 000 node randomizations of each observed network.
